# ALB-dNLR Score Predicts Mortality in Coronary Artery Disease Patients After Percutaneous Coronary Intervention

**DOI:** 10.3389/fcvm.2022.709868

**Published:** 2022-03-15

**Authors:** Wen-Juan Xiu, Hai-Tao Yang, Ying-Ying Zheng, Ting-Ting Wu, Xian-Geng Hou, Zhi-Hui Jiang, Yi Yang, Yi-Tong Ma, Xiang Xie

**Affiliations:** ^1^Department of Cardiology, First Affiliated Hospital of Xinjiang Medical University, Urumqi, China; ^2^Key Laboratory of Cardiac Injury and Repair of Henan Province, Department of Cardiology, First Affiliated Hospital of Zhengzhou University, Zhengzhou, China

**Keywords:** albumin and the derived neutrophil to lymphocyte ratio score (ALB-dNLR score), coronary artery disease, percutaneous coronary intervention, mortality, all-cause mortality, cardiac mortality, major adverse cardiovascular events, major adverse cardiovascular and cerebrovascular events

## Abstract

**Background:**

The influence of the albumin/derived neutrophil and lymphocyte ratio (ALB-dNLR) on the outcomes of patients with coronary artery disease (CAD) after percutaneous coronary intervention (PCI) is not known. Here, we aimed to determine the association between the ALB-dNLR score and post-PCI CAD patient outcomes.

**Methods:**

A total of 6,050 patients from the First Affiliated Hospital of Xinjiang Medical University were enrolled between January 2008 and December 2016. These patients were divided into three groups according to their ALB-dNLR scores (0 points, *n* = 1,121; 1 point, *n* = 3,119; 2 points, *n* = 1,810). Mortality after PCI [all-cause (ACM) and cardiac (CM)] was taken as the primary endpoint. The prognostic value of the ALB-dNLR score was determined with the Cox proportional hazard model after adjustment for covariates.

**Results:**

The ACM and CM rates differed among participants in the three groups (*P* = 0.007 and *P* = 0.034, respectively). Multivariate Cox analysis showed that the ALB-dNLR score independently predicted both ACM [1 point vs. 0 points, HR = 1.249 (95% CI: 0.79–1.774), *P* = 0.215; 2 points vs. 0 points, HR = 1.777 (95% CI: 1.239–2.549), *P* = 0.002] and CM [1 point vs. 0 points, HR = 1.294 (95% CI: 0.871–1.922), *P* = 0.202; 2 points vs. 0 points, HR = 1.782 (95% CI: 1.185–1.782), *P* = 0.027]. We also found that among male patients in the three groups, both ACM and CM rates differed (*P* = 0.006 and *P* = 0.017, respectively). Multivariate Cox analysis showed that the ALB-dNLR score independently predicted both ACM [1 point vs. 0 points, HR = 1.237 (95% CI: 0.806–0.330), *P* = 0.330; 2 points vs. 0 points, HR = 1.790 (95% CI: 1.159–2.764), *P* = 0.009] and CM [1 point vs. 0 points HR = 1.472 (95% CI: 0.892–2.430), *P* = 0.130; 2 points vs. 0 points, HR = 1.792 (95% CI: 1.182–3.289), *P* = 0.009].

**Conclusion:**

The ALB-dNLR score is a credible predictor for mortality in patients with CAD who have undergone PCI.

## Introduction

Coronary artery disease (CAD) has high morbidity and mortality rates (1, 2). Factors involved in CAD pathogenesis include inflammation (3, 4), lipids (5, 6), and dysfunctional fibrinolysis and coagulation (7, 8). As a potential inflammatory biomarker, leukocytes have been widely recognized in assessing the risk of cardiovascular disease (9). At present, the neutrophil/lymphocyte ratio (NLR) in leukocyte subtypes has been studied as a sensitive indicator of inflammation and has become an independent predictor of cardiovascular disease (10, 11). The NLR has proven useful in the risk stratification of cardiovascular patients and is the strongest predictor of prognosis in coronary artery bypass and PCI patients (12, 13). Horne et al. (14) first proposed a significant role for NLR in stable CAD patients. Although the total white blood cell count is known to predict myocardial infarction and death in CAD patients, high neutrophil counts or low lymphocyte counts have higher predictive power, and the strongest predictor of risk is the ratio of neutrophils to lymphocytes. They believe that white blood cell differential counts are similar to high-sensitivity C-reactive protein in their capacity to be used to predict risk and is even higher than hypersensitive C-reactive protein in this regard (14).

Systemic inflammation influences the synthesis of albumin, and the level of this serum protein is thus useful as an inflammatory marker. Albumin is synthesized in the liver and is involved in the maintenance of plasma colloid osmotic pressure, metabolic material transport, and nutrition. Studies have reported that as the serum albumin concentration decreases, the incidence of cardiovascular diseases increases (15). Albumin also has an antioxidant role in host defense (16, 17). The derived neutrophil to lymphocyte ratio (dNLR) is defined as neutrophils/(leukocytes-neutrophils) (18). The degree of systemic inflammation is indicated by the numbers of circulating immune cells, including neutrophils, lymphocytes, and monocytes (19). Albumin serves as a negative acute-phase reactant, and the circulatory level of albumin decreases as a result of increased transcapillary leakage or reduced hepatic synthesis mediated by interleukin-6 and tumor necrosis factor alpha. Therefore, a low albumin level may reflect an underlying inflammatory state, which increases the risk of mortality in various disease processes, including coronary artery disease. Alternatively, hypoalbuminemia may occur in the presence of coexisting nephrotic syndrome or chronic kidney disease, which are also risk factors for mortality (20).

The albumin level may reflect an underlying inflammatory and nutritional status. The derived neutrophil-to-lymphocyte ratio (dNLR) is defined as neutrophils/(leukocytes-neutrophils) and can reflect the severity of systematic inflammation. Previous research has demonstrated that systemic inflammation and malnutrition might contribute to the progression of CAD. The ALB-dNLR score consists of the serum albumin and derived neutrophil to lymphocyte ratio, indicating nutritional status and inflammatory conditions that are associated with the disease activity of CAD patients, while there is no investigation about the associations between ALB-dNLR score and disease activity in CAD patients.

Here, we retrospectively analyzed 6,050 post-PCI CAD patients to determine the value of the ALB-dNLR score in predicting clinical outcomes over long-term follow-up period.

## Methods

### Participants

**Figure 1:**
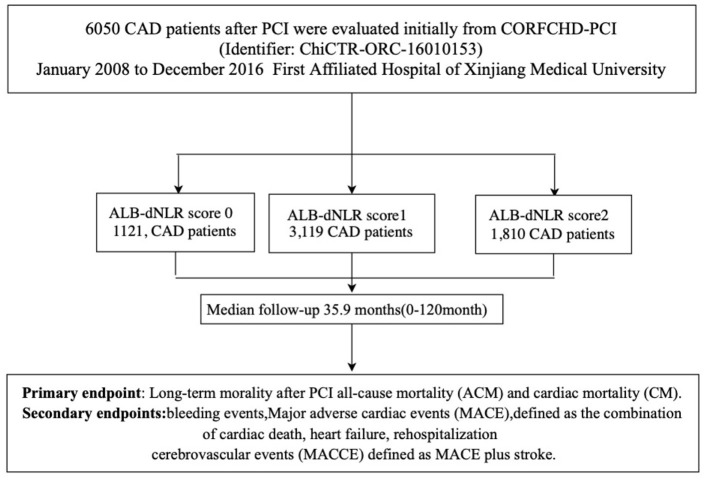
Flow chart of participants inclusion.

Participants were from the CORFCHD-PCI cohort (a large single-center retrospective cohort) comprising 6,050 CAD patients who had undergone PCI at the First Affiliated Hospital of Xinjiang Medical University from January 2008 to December 2016 (21). This information is registered at http://www.chictr.org.cn (identifier: ChiCTR-ORC-16010153). The selection of study subjects had strict inclusion/exclusion criteria. The inclusion criteria were acute ST-segment elevation myocardial infarction (STEMI), non-ST-segment elevation acute coronary syndrome (NSTE-ACS), and stable angina patients who had undergone coronary angiography and had at least one implanted stent. The exclusion criteria were malignant tumors, infectious diseases, hematological diseases, liver diseases, severe heart failure, congenital, rheumatic, or valvular heart disease, and severe kidney or liver dysfunction. Finally, we recruited 6,050 post-PCI CAD patients, including 1,121 patients with 0 points, 3,119 with 1 point and 1,810 with 2 points. The flowchart of the inclusion and exclusion criteria used in the selection of participants is shown in Figure 1.

The study protocol was approved by the Ethics Committee of the First Affiliated Hospital of Xinjiang Medical University and complies with the Declaration of Helsinki. As the study was a retrospective cohort study, it did not require the informed consent of patients.

### Data Collection

The collected data included demographics, blood and biochemical parameters, alcohol consumption, diabetes history, hypertension, smoking status, and family history of CAD. All patients underwent routine blood and blood biochemical index testing 24 h before coronary angiography. The inspection was completed by the Department of Laboratory Medicine, the First Affiliated Hospital of Xinjiang Medical University. According to the recommendations of the American Heart Association, hypertension was defined as a history of hypertension with active treatment or at least three resting blood pressure readings ≥140/90 mm Hg and at least two independent hygiene health care visits (22). The diabetes diagnosis was proposed by the WHO in 1999: ① symptoms of diabetes, intravenous plasma glucose ≥11.1 mmol/L (200 mg/dl) at any time, ② fasting venous plasma glucose ≥7.0 mmol/L (126 mg/dl), and ③ 2-h intravenous plasma glucose ≥11.1 mmol/L in an OGTT (75 g anhydrous glucose). Regarding the above three criteria, as long as one of the three is met, any one of the three items is checked again on the following day, and the check also meets the criteria, diabetes can be diagnosed (23). Hyperlipidemia was diagnosed according to the “Guidelines for the Prevention and Treatment of Dyslipidemia in Adults in China (2016).” Smoking status was classified as current, former, or never smoked with current smokers, including regular smoking over the previous 6 months. Similarly, people who had consumed alcohol in the previous 6 months were regarded as drinkers (24).

### dNLR and ALB-dNLR

Our study quotes the definition of dNLR and ALB-dNLR score used in Chen et al.'s (25) study. Briefly, the dNLR is defined as neutrophils/(leukocytes-neutrophils). Cutoff values from the ROC curve in Chen et al. (25) for dNLR and serum albumin were set at 1.37 and 37.6 g/L, respectively. However, the cutoff value of either serum albumin or dNLR was different from that in Chen et al.'s study. In our study, the cutoff values of the ROC curves for dNLR and serum albumin were 1.365 and 39.6 g/L, respectively. According to each cutoff value of serum albumin and dNLR, the patients were divided into three groups: patients with ALB-dNLR scores of 2, including participants with hypoalbuminemia ( ≤ 39.6 g/L) and high dNLR (>1.365); ALB-dNLR score of 1, including patients with either hypoproteinemia or high dNLR, and scores of 0 for patients without hypoproteinemia or high dNLR.

### Endpoints

Mortality during the 10-year follow-up was taken as the primary endpoint. This included both all-cause mortality (ACM) and cardiac mortality (CM). The secondary endpoints were major adverse cardiac events (MACEs), major adverse cardiac and cerebrovascular events (MACEs), and bleeding events. MACEs included cardiac death, heart failure, reinfarction and rehospitalization. All-cause death included both cardiac death and non-cardiac death. Death was classified as all-cause death if there was a clear non-cardiac cause of death; otherwise, death was considered to be cardiac death. Stroke included a sudden onset of dizziness, dysarthria, numbness, or aphasia as a result of cerebrovascular disease (caused by bleeding, thrombosis, embolism, or aneurysm rupture) of more than 24 h (26). Reinfarction refers to acute MI that occurs within 28 days of an event or recurrent MI. When ST elevation is ≥0.1 mV or new pathological Q waves appear again in at least two consecutive leads, especially when ischemic symptoms persist for 20 min or more (27). HF was referred to as systolic HF in accordance with a previous description (28). These events were decided by a blinded committee composed of 4 cardiovascular physicians and a nurse from the CORFCHD-PCI research group. If there are disagreements in the adjudication of the events, it will be solved according to the principle of the minority obeying the majority.

### Follow-Up

All post-PCI patients were followed up by six assessments over 0–120 months. Assessments were either office visits or by telephone.

### Statistical Analyses

Data were analyzed using SPSS version 22.0. Continuous variables are expressed as the means ± standard deviations, and categorical variables are expressed as frequencies and percentages. Analysis used the *t-test* for normally distributed variables; non-normally distributed data were analyzed by the Mann–Whitney *U* test or Kruskal–Wallis analysis of variance. Categorical variables were investigated by the χ-square test, and the cumulative incidence of major adverse events was analyzed by Kaplan–Meier analysis. Multivariable analysis was performed to assess the prognostic value of the ALB-dNLR and adverse outcomes after adjusting for confounders, such as sex, age, estimated glomerular filtration rate (eGFR), total cholesterol (TC), triglycerides (TGs), chronic total occlusion lesions (CTOs), multivessel lesions (MLs), and stains, and to calculate hazard ratios (HRs) and 95% confidence intervals (CIs). *P* <0.05 was considered significant.

## Results

### Baseline Data

The optimal cutoff value (dNLR = 1.365 and serum albumin = 39.6 g/L) was defined according to the receiver operating curve (ROC). The patients were divided into three groups according to ALB-dNLR scores: 0 points (*n* = 1,121), 1 point (*n* = 3,119), and 2 points (*n* = 1,810). Many variables were significantly different among the three groups. As shown in Table 1, compared with the other two groups, in the ALB-dNLR 2-point group, there were more men (*p* < 0.001), older age (*p* = 0.016), higher creatinine (Cr) (*p* < 0.001), higher WBC (*p* < 0.001), higher neutrophils (*p* < 0.001), and higher BUN (*p* < 0.001). Albumin (*p* < 0.001), lymphocytes (*p* < 0.001), estimated glomerular filtration rate (eGFR) (*p* = 0.038), triglycerides (TGs) (*p* = 0.009), total cholesterol (TC) (*p* < 0.001), low-density lipoprotein cholesterol (LDL-C) (*p* < 0.001) and ejection fraction (*p* < 0.001) were significantly lower than those in the other two groups. Calcium channel blockade (CCB) (*p* = 0.035) and statins (*p* = 0.009) were also significantly different among the three groups. Other baseline data did not differ (all *P* > 0.05). As shown in Table 2, the characteristics of chronic total occlusion (CTO) (*P* = 0.046), multivessel lesions (MLs) (*P* = 0.020) and the number of lesional vessels (*P* = 0.019) differed significantly among the three groups.

**Table 1:** Characteristics of participants of the three groups.

**Variables**	**ALB-dNLR score**				
	**0**	**1**	**2**	**Chi-square or *t***	***p*-Value**
Sex, male, *n* (%)	770 (68.7)	2,335 (74.9)	1,394 (77)	26.491	**< 0.001**
Smoking, *n* (%)	438 (39.1)	1,262 (40.5)	721 (39.8)	0.668	0.716
Alcohol drinking, *n* (%)	323 (28.8)	935 (30.0)	509 (28.1)	2.000	0.368
Diabetes, *n* (%)	273 (24.4)	748 (24.0)	430 (23.8)	0.145	0.930
Hypertension, *n* (%)	447 (39.9)	1,315 (42.2)	794 (43.9)	4.462	0.107
Family CAD history, *n* (%)	147 (13.1)	377 (12.1)	236 (13.0)	1.338	0.512
Age, years	58.76 ± 10.71	59.49 ± 10.88	59.95 ± 10.81	4.122	**0.016**
SBP, mm Hg	127.03 ± 18.28	126.84 ± 18.87	127.34 ± 18.90	0.406	0.666
DBP, mm Hg	76.64 ± 11.42	76.19 ± 11.11	76.30 ± 11.56	0.661	0.517
WBC, 10^9^/L	6.55 ± 1.08	7.37 ± 2.40	7.84 ± 2.62	103.071	**<0.001**
Neutrophil, 10^9^/L	3.34 ± 1.15	4.61 ± 2.23	5.43 ± 2.39	330.634	**<0.001**
Lymphocyte, 10^9^/L	2.42 ± 0.76	2.02 ± 0.77	1.74 ± 0.65	291.721	**<0.001**
Monocyte, 10^9^/L	6.55 ± 1.08	7.37 ± 2.40	7.84 ± 2.62	2.065	**0.074**
Albumin, g/L	39.81 ± 11.41	37.85 ± 9.87	35.40 ± 4.42	89.268	**<0.001**
BUN, mmol/L	5.48 ± 1.49	5.49 ± 1.64	5.58 ± 1.84	15.712	**<0.001**
UA, mmol/L	321.29 ± 88.42	323.88 ± 89.69	323.51 ± 92.02	0.330	0.719
Cr, umol/L	72.91 ± 17.33	76.15 ± 19.86	77.27 ± 22.76	−1.992	**<0.001**
eGFR, ml/min	99.17 ±26.22	96.60 ± 27.27	96.55 ± 35.74	3.265	**0.038**
TG, mmol/L	2.03 ± 1.26	1.88 ± 1.25	1.84 ± 1.28	7.932	**<0.001**
TC, mmol/L	4.07 ± 1.12	3.96 ± 1.11	3.88 ± 1.06	9.019	**<0.001**
LDL-C, mmol/L	2.55 ± 0.95	2.47 ± 0.92	2.37 ± 0.86	13.440	**<0.001**
HDL-C, mmol/L	1.06 ± 0.51	1.01 ± 0.46	1.00 ± 0.48	3.083	0.050
Ejection fraction, %	62.06 ± 6.35	61.12 ± 6.90	60.36 ± 7.57	18.165	**<0.001**
Lp(a), mmol/L	217.01 ± 179.26	219.85 ± 179.02	222.84 ± 171.54	0.367	0.693
ACEI or ARB, *n* (%)	240 (21.5)	725 (23.4)	402 (22.3)	1.981	0.371
β-blocker, *n* (%)	451 (40.3)	1,247 (40.2)	731 (40.5)	0.041	0.980
CCB, *n* (%)	127 (11.4)	385 (12.4)	180 (10.0)	6.687	**0.035**
Statins, *n* (%)	566 (50.8)	1,727 (54.9)	966 (53.7)	9.519	**0.009**
**Male**					
Smoking, *n* (%)	427 (55.5)	1,225 (52.5)	702 (50.4)	5.338	0.069
Alcohol drinking, *n* (%)	305 (39.7)	891 (38.2)	495 (34.8)	6.304	**0.043**
Diabetes, *n* = (%)	184 (23.9)	536 (23.0)	316 (22.7)	0.459	0.795
Hypertension, *n* (%)	285 (37.1)	902 (38.6)	587 (42.1)	6.622	**0.036**
Family CAD history, *n* (%)	105 (13.7)	288 (12.3)	178 (12.8)	0.920	0.631
Age, years	56.80 ± 10.67	58.02 ± 10.98	58.77 ± 111.02	8.017	**<0.001**
SBP, mm Hg	126.10 ± 18.25	125.62 ± 18.41	126.244 ± 18.60	0.557	0.573
DBP, mm Hg	77.40 ± 11.65	76.26 ± 11.58	76.45 ± 11.41	3.165	**0.042**
WBC, 10^9^/L	6.72 ± 1.77	7.52 ± 2.42	8.00 ± 2.69	69.079	**<0.001**
Albumin, g/L	40.01 ± 11.27	37.98 ± 9.83	35.33 ± 4.44	76.445	**<0.001**
BUN, mmol/L	5.59 ± 1.47	5.55 ± 1.63	5.65 ± 1.80	1.445	0.223
UA, mmol/L	337.58 ± 86.80	335.13 ± 88.04	334.29 ± 89.05	0.330	0.236
Cr, umol/L	76.37 ± 16.68	79.67 ± 19.06	79.69 ± 22.23	8.511	**<0.001**
eGFR, ml/min	102.99 ± 26.22	98.58 ± 27.29	99.08 ± 28.74	7.248	**0.001**
TG, mmol/L	2.05 ± 1.24	1.86 ± 1.18	1.84 ± 1.28	7.632	**<0.001**
TC, mmol/L	4.04 ± 1.14	3.95 ±1.09	3.88 ± 1.06	4.545	**0.001**
LDL-C, mmol/L	2.53 ± 0.93	2.47 ± 0.91	2.37 ± 0.86	7.992	**<0.001**
HDL-C, mmol/L	1.05 ± 0.55	1.00 ± 0.42	1.00 ± 0.53	3.157	**0.043**
Ejection fraction	62.10 ± 6.41	61.11 ± 6.84	60.13 ± 7.74	17.827	**<0.001**
Lp(a), mmol/L	213.49 ± 172.79	216.83 ± 176.63	224.32 ± 175.72	1.121	0.326
ACEI or ARB, *n* (%)	171 (22.3)	546 (23.5)	311 (22.4)	0.833	0.659
β-blocker, *n* (%)	311 (40.5)	957 (41.2)	566 (40.7)	0.117	0.943
CCB, *n* (%)	84 (11.0)	289 (12.4)	139 (10.0)	6.687	0.072
Statins, *n* (%)	395 (51.6)	1,323 (57.3)	764 (55.2)	7.597	**0.022**
**Female**					
Smoking, *n* (%)	11 (3.1)	37 (4.7)	19 (4.6)	1.559	0.459
Alcohol drinking, *n* (%)	18 (5.1)	44 (5.6)	524 (5.5)	0.163	0.922
Diabetes, *n* (%)	89 (25.4)	212 (27.0)	114 (27.4)	0.473	0.790
Hypertension, *n* (%)	162 (46.2)	413 (52.7)	207 (49.8)	4,228	0.121
Family CAD history, *n* (%)	42 (12.0)	89 (11.4)	58 (13.9)	1.725	0.422
Age, years	63.06 ± 9.48	63.84 ± 9.28	63.88 ± 9.02	1.001	0.368
SBP, mm Hg	129.09 ± 18.22	130.48 ± 19.73	131.02 ± 19.44	1.036	0.355
DBP, mm Hg	74.98 ± 10.74	75.96 ± 10.78	76.46 ± 11.50	1.791	0.167
WBC, 10^9^/L	6.16 ± 1.82	6.93 ± 2.28	7.32 ± 2.29	27.429	**<0.001**
Albumin, g/L	39.38 ± 11.71	37.45 ± 9.97	35.64 ± 4.33	89.268	**<0.001**
BUN, mmol/L	5.25 ± 1.50	5.31 ± 1.69	5.37 ± 1.95	0.469	0.626
UA, mmol/L	285.46 ± 81.19	290.60 ± 86.82	291.52 ± 85.81	2.075	0.126
Cr, umol/L	65.30 ± 16.30	65.74 ± 18.48	69.23 ± 22.71	5.369	**0.005**
eGFR, ml/min	90.78 ± 24.21	90.73 ± 26.17	88.15 ± 51.89	0.831	0.436
TG, mmol/L	2.01 ± 1.31	1.94 ± 1.46	1.86 ± 1.27	1.014	0.363
TC, mmol/L	4.14± 1.22	4.01 ±1.15	3.88 ± 1.03	4.507	**0.011**
LDL-C, mmol/L	2.60 ± 0.99	2.47 ± 0.94	2.36 ± 0.89	**5.812**	**0.003**
HDL-C, mmol/L	1.07 ± 0.42	1.05 ± 0.57	097 ± 0.27	4.867	**0.008**
Ejection fraction	61.97 ± 6.25	61.16 ± 7.06	61.13 ± 6.94	1.760	0.172
Lp(a), mmol/L	224.93 ± 193.10	228.76 ± 185.72	217.96 ± 157.14	0.473	0.623
ACEI or ARB, *n* (%)	69 (19.7)	179 (23.1)	91 (22.0)	1.637	0.441
β-blocker, *n* (%)	140 (39.9)	290 (37.4)	165 (39.8)	0.936	0.626
CCB, *n* (%)	44 (12.3)	96 (12.4)	41 (9.9)	1.748	0.409
Statins, *n* (%)	171 (48.9)	404 (52.3)	202 (48.9)	1.753	0.416

*ACEI, angiotensin-converting enzymein hibitor; ApoA1, apolipoprotein A1; ApoB, apolipoprotein B; ARB, angiotensin receptor blocker; BUN, blood urea nitrogen; CCB, calcium channel blocker; Cr, creatinine; DBP, diastolic blood pressure; eGFR, estimated glomerular filtration rate; HDL-C, high- density lipoprotein cholesterol; LDL-C, low-density lipoprotein cholesterol; Lp(a), lipoprotein a; SBP, systolic blood pressure; TC, total cholesterol; TG, triglyceride; UA, uric acid; WBC, white blood cell. The bold values indicates p-value <0.05*.

**Table 2:** Baseline treatments and procedure characteristics.

**Variables**	**ALB-dNLR score**				
	**0**	**1**	**2**	**Chi-square or *t***	***p*-Value**
DES, *n* (%)	1,055 (94.1)	2,928 (93.9)	1,717 (94.9)	1.959	0.376
CTO, *n* (%)	236 (21.1)	725 (23.3)	453 (25.0)	6.162	**0.046**
LM, *n* (%)	77 (6.9)	209 (6.7)	147 (8.1)	3.641	0.162
ML, *n* (%)	734 (65.5)	1,975 (63.3)	1,217 (67.2)	7.829	**0.020**
No. of lesional vessel	2.01 ± 0.83	1.98 ± 0.84	2.05 ± 0.84	3.986	**0.019**
Length of stents, mm	28.02 ± 6.98	27.98 ± 6.95	27.95 ± 6.99	0.338	0.957
No. of stents	1.05 ± 0.23	1.03 ± 0.21	1.04 ± 0.22	2.508	0.128
Expansion pressure	11.85± 2.58	11.87 ± 3.09	11.81 ± 2.48	0.140	0.869
Post-expansion pressure	13.95 ± 4.05	13.86 ± 3.39	13.86 ± 3.43	0.211	0.809
**Male**					
DES, *n* (%)	724 (94)	2,191 (93.8)	1,320 (94.7)	1.184	0.553
CTO, *n* (%)	166 (21.6)	559 (23.9)	366 (26.3)	6.211	**0.045**
LM, *n* (%)	55 (7.1)	160 (6.9)	120 (8.6)	4.030	0.133
ML, *n* (%)	500 (64.9)	1,494 (64)	1,394 (100)	7.549	**0.023**
No. of lesional vessel	2.01 ± 0.84	1.99 ± 0.84	2.07 ± 1.83	3.909	**0.020**
Length of stents, mm	28.06 ± 6.90	27.92 ± 6.92	27.92 ± 7.03	0.122	0.885
No. of stents	1.05 ± 0.23	1.03 ± 0.20	1.04 ± 0.23	2.550	0.078
Expansion pressure	11.77 ± 2.49	11.86 ± 2.52	11.80 ± 2.52	0.230	0.794
Post-expansion pressure	13.95 ± 3.26	13.84 ± 3.37	13.83 ± 3.48	0.251	0.778
**Female**					
DES, *n* (%)	332 (94.3)	737 (94.1)	397 (95.4)	0.936	0.626
CTO, *n* (%)	70 (19.9)	166 (21.2)	87 (20.9)	0.234	0.889
LM, *n* (%)	22 (6.3)	49 (6.3)	27 (6.5)	0.027	0.987
ML, *n* (%)	234 (66.7)	481 (61.4)	264 (163.5)	2.878	0.237
No. of lesional vessel	2.01 ± 0.82	1.96 ± 0.85	2.00 ± 0.85	0.600	0.549
Length of stents, mm	27.96 ± 7.16	28.15 ± 7.04	28.05 ± 6.86	0.099	0.905
No. of stents	1.04 ± 0.25	1.04 ± 0.24	1.03 ± 0.23	0.174	0.840
Expansion pressure	12.04 ± 2.80	11.92 ± 4.29	11.85 ± 2.34	0.125	0.882
Post-expansion pressure	13.96 ± 5.36	13.93 ± 3.46	13.98 ± 3.26	0.019	0.981

*ALB-dNLR score, albumin and the derived neutrophil to lymphocyte ratio score; CTO, chronic total occlusion lesions; LM, left main disease; ML, multi-vessel lesions; DES, drug-eluting stent. The bold values indicates p-value <0.05*.

As shown in Tables 1, 2, among male patients, alcohol consumption, hypertension, age, DBP, WBC, albumin, Cr, eGFR, TG, TC, LDL-C, HDL-C, EF, statins, CTO, ML, and the number of lesional vessels were significantly different among the three groups (all *p* <0.05). Among female patients in the three groups, WBC, albumin, Cr, TC, LDL-C, and HDL-C were also significantly different (all *p* < 0.05).

### Clinical Outcomes

#### All-Cause Mortality

After a median follow-up period of 35.9 months, ACM occurred more frequently in the ALB-dNLR score 2 group [116 (6.4%)] than in the other two groups [0 points, 45 (4.0%) and 1 point, 1,481 (4.7%) (*p* = 0.007)], as shown in Table 3.

**Table 3:** Outcomes comparison among three groups.

**Outcomes**	**ALB-dNLR score**				
	**0**	**1**	**2**	**Chi-square**	***p*-Value**
ACM, *n* (%)	45 (4.0)	148 (4.7)	116 (6.4)	0.934	**0.007**
CM, *n* (%)	36 (3.2)	123 (3.9)	92 (5.1)	6.779	**0.034**
MACCEs, *n* (%)	159 (14.2)	418 (13.4)	285 (13.4)	5.516	0.076
MACEs, *n* (%)	148 (13.2)	377 (12.1)	260 (14.4)	5.324	0.070
Heart failure, *n* (%)	32 (2.9)	85 (2.7)	64 (3.5)	2.683	0.261
Stroke, *n* (%)	11 (1.1)	46 (1.5)	25 (1.4)	1.515	0.469
Bleeding events, *n* (%)	35 (3.1)	99 (3.2)	41 (2.3)	3.627	0.163
Rehospitalizationl, *n* (%)	163 (14.5)	415 (13.3)	241 (13.3)	1.184	0.553
**Male**					
ACM, *n* (%)	28 (3.6)	107 (4.6)	90 (6.5)	10.093	**0.006**
CM, *n* (%)	20 (2.6)	92 (3.9)	71 (5.1)	8.121	**0.017**
MACCEs, *n* (%)	113 (14.7	318 (13.6)	223 (16)	3.989	0.136
MACEs, *n* (%)	102 (13.2)	290 (12.4)	204 (14.6)	3.725	0.155
**Female**					
ACM, *n* (%)	17 (4.8)	41 (5.2)	26 (6.3)	0.843	0.656
CM, *n* (%)	16 (4.6)	31 (4.0)	21 (5.0)	0.809	0.667
MACCEs, *n* (%)	46 (13.1)	100 (12.8)	62 (14.9)	1.117	0.572
MACEs, *n* (%)	46 (13.1)	87 (11.1)	56 (13.5)	1.779	0.411

*ALB-dNLR score, albumin and the derived neutrophil to lymphocyte ratio score; ACM, all-cause mortality; CM, cardiac mortality; MACE, major adverse cardiovascular event; MACCE, major adverse cardiovascular and cerebrovascular event; %, relative frequencies. The bold values indicates p-value < 0.05*.

Univariate analysis showed that the risk of all-cause death increased 72.1% in the ALB-dNLR score 2-point group [adjusted HR = 1.721 (1.220–2.429), *P* = 0.002] compared to the 0-point group (Table 4).

**Table 4:** Univariate logistic regression analysis of ACM, cardiac death, MACCEs, and MACEs.

**Characteristics**	**ACM**		**CM**		**MACCEs**		**MACEs**	
	**HR (95% CI)**	***p*-Value**	**HR (95% CI)**	***p*-Value**	**HR (95% CI)**	***p*-Value**	**HR (95% CI)**	***p*-Value**
ALB-dNLR score 0	1		1		1		1	
ALB-dNLR score 1	1.249 (0.894–1.744)	0.192	1.298 (0.895–1.883)	0.168	0.999 (0.833–1.200)	0.996	0.968 (0.800–1.171)	0.737
ALB-dNLR score 2	1.721 (1.220–2.429)	**0.002**	1.706 (1.161–2.509)	**0.007**	1.195 (0.975–1.466)	0.061	1.179 (0.964–1.443)	0.110
**Male**								
ALB-dNLR score 0	1		1		1		1	
ALB-dNLR score 1	1.310 (0.864–1.986)	0.204	1.578 (0.973–2.559)	0.065	0.968 (0.833–1.200)	0.763	0.976 (0.779–1.223)	0.835
ALB-dNLR score 2	1.879 (1.229–2.871)	**0.004**	2.708 (1.265–3.414)	**0.004**	1.163 (0.928–1.459)	0.190	1.178 (0.928–1.494)	0.178
**Female**								
ALB-dNLR score 0	1		1		1		1	
ALB-dNLR score 1	1.221 (0.692–2.154)	0.490	0.977 (0.533–1.790)	0.939	1.071 (0.755–1.521)	0.700	0.934 (0.652–1.336)	0.708
ALB-dNLR score 2	1.8516 (0.822–2.799)	0.183	1.288 (0.671–2.472)	0.447	1.316 (0.898–1.929)	0.159	1.189 (0.804–1.757)	0.386

*ALB-dNLR score, albumin and the derived neutrophil to lymphocyte ratio score; ACM, all-cause mortality; CM, cardiac mortality; MACE, major adverse cardiovascular event; MACCE, major adverse cardiovascular and cerebrovascular event; %, relative frequencies. The bold values indicates p-value < 0.05*.

**Figure 2:**
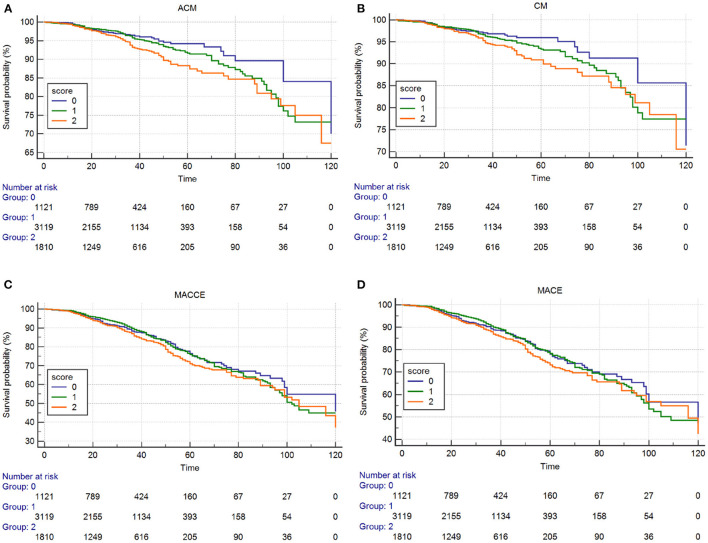
Cumulative Kaplan–Meier estimates of the time to the first adjudicated occurrence of primary endpoint and secondary endpoints. **(A)** ACM; **(B)** CM; **(C)** MACCEs; **(D)** MACE.

Kaplan–Meier and log-rank analyses showed a significant gradual increased risk in the high ALB-dNLR group, which indicates that its long-term mortality gradually increased with increasing ALB-dNLR score (*P* = 0.0025, Figure 2). Multivariate analysis evaluated the prognostic value of ALB-dNLR after adjustment for age, sex, eGFR, TG, TC, statins, CTO, and ML. The multivariable Cox regression model showed that the ALB-dNLR score was independently predictive of ACM in post-PCI CAD patients. The risk of mortality increased 77.7% in the ALB-dNLR score 2-point group [adjusted HR = 1.777 (1.239–2.549), *P* = 0.002] compared to patients in the ALB-dNLR score 0 group (Table 5). In addition, compared to a score of 0, the risk of patients with a score of 1 increased by 24.9% [adjusted HR = 1.249 (0.79–1.774), *P* = 0.215].

**Table 5:** Cox proportional hazards analysis of ACM, cardiac death, MACCEs, and MACEs.

**Characteristics**	**ACM**		**CM**		**MACCEs**		**MACEs**	
	**HR (95% CI)**	***p*-Value**	**HR (95% CI)**	***p*-Value**	**HR (95% CI)**	***p*-Value**	**HR (95% CI)**	***p*-Value**
Age	1.028 (1.016–1.040)	**0.000**	1.019 (1.006–1.033)	**0.004**	1.004 (0.997–1.011)	0.283	1.001 (0.994–1.008)	0.736
Sex	0.957 (0.732–1.252)	0.749	0.970 (0.717–1.312)	0.843	0.970 (0.819–1.149)	0.725	0.980 (0.820–1.171)	0.822
eGFR	0.998 (0.993–1.003)	0.366	0.996 (0.991–1.002)	0.185	1.000 (0.998–1.003)	0.815	1.001 (0.998–1.004)	0.518
TG	1.023 (0.939–1.115)	0.603	0.998 (0.900–1.107)	0.687	1.014 (0.958–1.073)	0.628	0.997 (0.938–1.059)	0.919
TC	0.994 (0.890–1.110)	0.913	1.039 (0.920–1.174)	0.539	0.952 (0.890–1.018)	0.152	0.967 (0.901–1.038)	0.352
CTO	1.592 (1.226–2.066)	**0.000**	1.709 (1.281–2.280)	**0.000**	1.266 (1.077–1.488)	**0.004**	1.315 (1.112–1.554)	**0.001**
ML	1.384 (1.055–1.816)	**0.019**	1.396 (1.029–1.895)	**0.032**	1.292 (1.104–1.511)	**0.001**	1.346 (1.139–1.589)	**0.000**
Stains	0.104 (0.070–0.154)	**0.000**	0.121 (0.08–0.184)	**0.000**	0.831 (0.723–0.956)	**0.001**	0.783 (0.676–0.907)	**0.001**
ALB-dNLR score 0	1		1		1		1	
ALB-dNLR score 1	1.249 (0.79–1.774)	0.215	1.294 (0.871–1.922)	0.202	0.998 (0.823–1.210)	0.981	0.983 (0.803–1.202)	0.864
ALB-dNLR score 2	1.777 (1.239–2.549)	**0.002**	1.782 (1.185–2.680)	**0.006**	1.195 (0.975–1.466)	0.087	1.194 (0.965–1.478)	0.102
**Male**								
Age	1.036 (1.022–1.050)	**0.000**	1.030 (1.015–1.045)	**0.000**	1.010 (1.003–1.018)	0.008	1.007 (0.999–1.015)	0.088
eGFR	1.000 (0.995–1.006)	0.852	1.000 (0.994–1.005)	0.885	1.002 (0.999–1.005)	0.153	1.003 (1.000–1.006)	0.056
TG	1.008 (0.902–1.126)	0.892	0.946 (0.826–1.084)	0.426	0.992 (0.924–1.065)	0.822	0.966 (0.894–1.043)	0.372
TC	0.998 (0.867–1.129)	0.871	1.020 (0.881–1.180)	0.793	0.972 (0.900–1.050)	0.470	0.982 (0.906–1.065)	0.662
CTO	1.555 (1.151–2.102)	**0.004**	1.647 (1.180–2.300)	0.003	1.236 (1.028–1.458)	**0.024**	1.283 (1.060–1.552)	**0.010**
ML	1.355 (0.986–1.861)	0.061	1.321 (0.927–1.881)	0124	1.224 (1.024–1.463)	**0.026**	1.261 (1.045–1.552)	**0.016**
Stains	0.095 (0.059–0.152)	**0.000**	0.106 (0.064–0.175)	**0.000**	0.811 (0.691–0.952)	**0.010**	0.644 (0.723–0.901)	**0.002**
ALB-dNLR score 0	1		1		1		1	
ALB-dNLR score 1	1.237 (0.806–1.899)	0.330	1.472 (0.892–2.430)	0.130	0.950 (0.759–1.189)	0.655	0.979 (0.773–1.240)	0.861
ALB-dNLR score 2	1.790 (1.159–2.764)	**0.009**	1.972 (1.182–3.289)	**0.009**	1.105 (0.872–1.399)	0.409	1.143 (0.891–1.466)	0.292
**Female**								
Age	0.996 (0.971–1.023)	0.790	0.978 (0.951–1.007)	0.132	0.974 (0.958–0.999)	**0.001**	0.975 (0.958–0.992)	0.151
eGFR	0.989 (0.978–0.999)	0.027	0.986 (0.974–0.997)	0.015	0.993 (0.987–0.999)	0.030	0.993 (0.986–0.999)	0.004
TG	1.017 (0.895–1.157)	0.793	0.985 (0.847–1.145)	0.842	1.039 (0.948–1.139)	0.416	1.036 (0.942–1.139)	0.027
TC	1.030 (0.836–1.268)	0.785	1.118 (0.889–1.405)	0.340	0.898 (0.781–1.033)	0.132	0.928 (0.802–1.073)	0.469
CTO	1.603 (0.953–2.726)	0.082	1.722 (0.961–3.087)	0.068	1.351 (0.959–1.904)	0.085	1.380 (0.966–1.971)	0.314
ML	1.603 (0.905–2.622)	0.112	1.726 (0.933–3.194)	0.082	1.633 (1.166–2.287)	**0.004**	1.755 (1.223–2.518)	0.077
Stains	1.540 (0.067–0.291)	**0.000**	0.184 (0.087–0.388)	**0.000**	0.994 (0.706–1.262)	0.698	0.895 (0.659–1.216)	0.480
ALB-dNLR score 0	1		1		1		1	
ALB-dNLR score 1	1.362 (0.735–2.626)	0.327	1.061 (0.545–2.067)	0.861	1.135 (0.777–1.660)	0.512	0.986 (0.668–1.454)	0.165
ALB-dNLR score 2	1.738 (0.899–3.360)	0.100	1.532 (0.754–3.110)	0.238	1.482 (0.988–2.222)	0.057	1.354 (0.895–2.049)	0.941

*ALB-dNLR score, albumin and the derived neutrophil to lymphocyte ratio score; eGFR, estimated glomerular filtration rate; CTO, chronic total occlusion lesions; ML, multi-vessel lesions; TC, total cholesterol; TG, triglyceride. The bold values indicates p-value < 0.05*.

Among male patients, ACM occurred more frequently in the ALB-dNLR score 2 group [107 (6.5%)] than in the other two groups [0 points, 28 (3.6%) and 1 point, 107 (4.7%) (*p* = 0.006)], as shown in Table 3.

Univariate analysis showed that the risk of all-cause death increased 87.9% in participants in the ALB-dNLR score 2-point group [adjusted HR = 1.879 (1.229–2.871), *P* = 0.004] compared to those in the 0-point group (Table 4).

**Figure 3:**
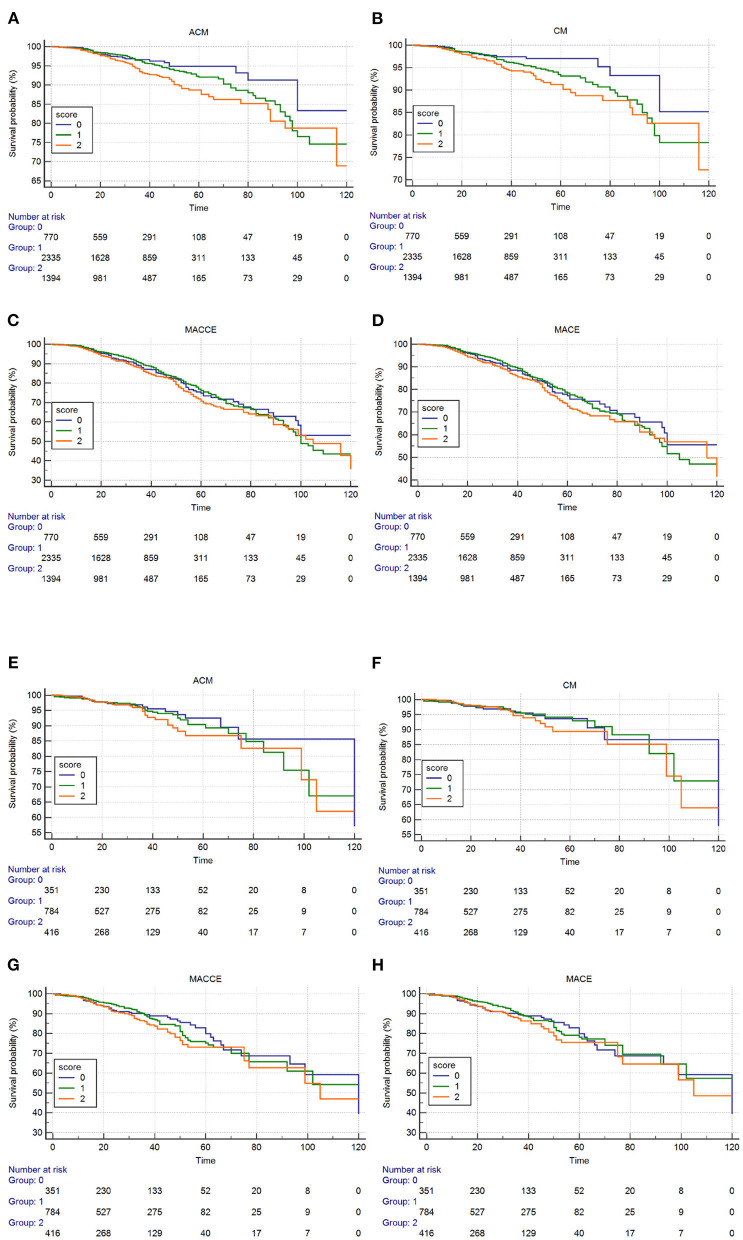
Cumulative Kaplan–Meier estimates of the time to the first adjudicated occurrence of primary endpoint and secondary endpoints. Male **(A–D)**: **(A)** ACM; **(B)** CM; **(C)** MACCEs; **(D)** MACE. Female **(E–H)**: **(E)** ACM; **(F)** CM; **(G)** MACCEs; **(H)** MACE.

The Kaplan–Meier curves and log-rank test demonstrated a significant difference in ACM in the three groups (*P* = 0.0036, Figure 3). After risk factor adjustment, the Cox proportional hazards model showed that the ALB-dNLR score was an independent prognostic factor for CAD patients after PCI. The risk of mortality increased 79% in the ALB-dNLR score 2-point group [adjusted HR = 1.790 (1.159–2.764), *P* = 0.009] compared to patients in the ALB-dNLR score 0 group (Table 5).

Among female patients, we did not find significant differences in ACM among the three groups. Univariate analysis and multivariable Cox regression showed that the ALB-dNLR score was not independently predictive of ACM in post-PCI CAD patients.

#### Cardiac Mortality

CM incidence also differed significantly [36 (3.2%) vs. 123 (3.9%) vs. 92 (5.1%), *P* = 0.034] among participants in the three groups. CM was more frequent in the ALB-dNLR score of 2 than in the other two groups (Table 3).

Univariate analysis showed that the risk of cardiac death increased 70.6% in participants in the ALB-dNLR score 2-point group [adjusted HR = 1.706 (1.161–2.509), *P* = 0.007] compared to participants in the score of 0 group (Table 4).

The Kaplan–Meier curves and log-rank test demonstrated a significant difference in CM among participants in the three groups (*P* = 0.014, Figure 2). After risk factor adjustment, the Cox proportional hazards model showed that the ALB-dNLR score was an independent prognostic factor for CAD patients after PCI. The risk of cardiac death increased 78.2% in participants in the ALB-dNLR score 2-point group [adjusted HR = 1.782 (1.185–2.680), *P* = 0.006] compared to participants in the 0-point group. TC, CTO, and multivessel lesions were found to be associated with ACM, CM, MACCEs, and MACEs after potential confounding factors were adjusted (all *P* < 0.05, Table 5). We did not find a significant difference between the score of 0 and the score of 1 in terms of CM (HR = 1.294, 95% CI: 0.871–1.922, *P* = 0.202).

Among male patients in the three groups, CM incidence also differed significantly [20 (2.6%) vs. 92 (3.9%) vs. 71 (5.1%), *P* = 0.017]. CM was more frequent in the ALB-dNLR score of 2 than in the other two groups (Table 3).

Univariate analysis showed that the risk of cardiac death increased 1-fold in the ALB-dNLR score 2-point group [adjusted HR = 2.708 (1.265–3.414), *P* = 0.004] compared to the 0-point group (Table 4).

The Kaplan–Meier curves and log-rank test demonstrated a significant difference in CM among the three groups (*P* = 0.010, Figure 3). After risk factor adjustment, the Cox proportional hazards model showed that the ALB-dNLR score was an independent prognostic factor for CAD patients after PCI. The risk of cardiac death increased 97.2% in the ALB-dNLR score 2-point group [adjusted HR = 1.972 (1.185–3.289), *P* = 0.009] compared to the 0-point group (Table 5).

Among female patients, we did not find significant differences in CM among the three groups. Univariate analysis and multivariable Cox regression showed that the ALB-dNLR score was not independently predictive of CM in post-PCI CAD patients.

#### MACCEs and MACEs

We did not found that the incidences of MACCEs [159 (14.2%) vs. 418 (13.4%) vs. 285 (13.4%), *P* = 0.076], MACEs [148 (13.2%) vs. 377 (12.1%) vs. 260 (14.4%), *P* = 0.070], HF [32 (2.9%) vs. 85 (2.7%) vs. 64 (3.5%), *P* = 0.261], bleeding events [35 (3.1%) vs. 99 (3.2%) vs. 41 (2.3%), *P* = 0.163], stroke [11 (1.1%) vs. 46 (1.5%) vs. 25 (1.4%), *P* = 0.469], and rehospitalization [163 (14.5%) vs. 415 (13.3%) vs. 241 (13.3%), *P* = 0.083] differed significantly among participants in the three groups (Table 3).

The Kaplan–Meier curves and log-rank test demonstrated no significant difference in MACCEs and MACEs (Figure 2) among the three groups. The multivariate Cox regression model showed that the MACCEs and MACEs were not significantly different among participants in the three groups (*P* > 0.05, Table 5).

Among male patients and female patients, we did not find that the incidences of MACCEs and MACEs differed significantly in the three groups (Table 3). The Kaplan–Meier curves and log-rank test demonstrated no significant difference in MACCEs and MACEs (Figure 2) among the three groups. Univariate analysis and multivariate Cox regression showed that the MACCEs and MACEs were not significantly different among participants in the three groups (*P* > 0.05, Tables 4, 5).

**Figure 4:**
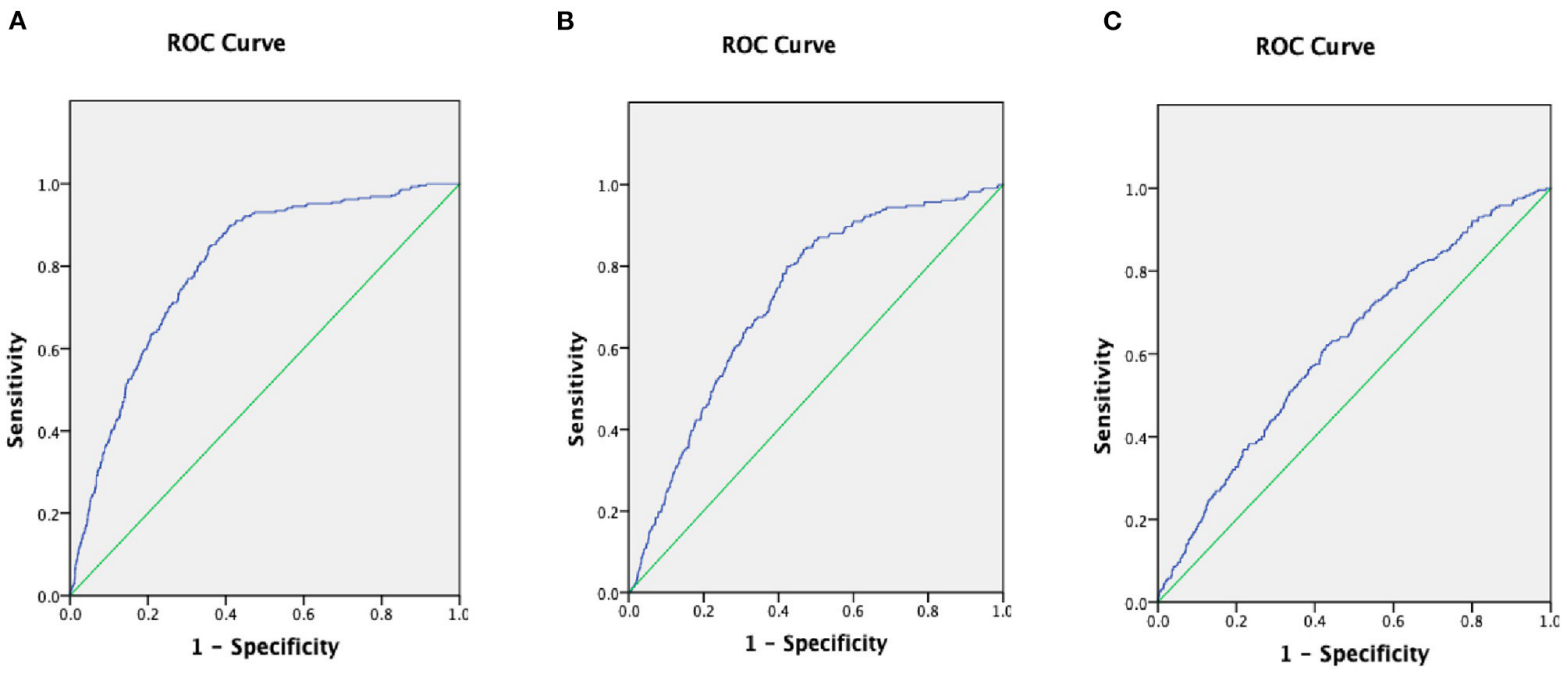
The areas under the curves (AUC) of AUC of ALB-dNLR +classic risk model **(A)**, the AUC of dNLR+ classic risk model **(B)**, and he AUC of ALB+ classic risk model **(C)** in CAD patients. ALB, albumin; dNLR, derived neutrophil to lymphocyte ratio.

To assess the diagnostic ability of the ALB-dNLR in CAD patients and compare it with classical risk predictors, we generated ROC curves and compared the area under the curve (AUC) values (Figure 4). The results showed that the AUC of the ALB-dNLR +classic risk model was 0.794 (95% CI: 0.771–0.817), the AUC of the dNLR+ classic risk model was 0.720 (95% CI: 0.690–0.750) and the AUC of the ALB+ classic risk model was 0.620 (95% CI: 0.588–0.652). Therefore, we believe that the predictive performance of ALB-dNLR is stronger than that of albumin or dNLR alone.

## Discussion

This retrospective study of 1,121 patients with an ALB-dNLR of 0 points, 3,119 with an ALB-dNLR of 1 point, and 1,810 with an ALB-dNLR of 2 points from the First Affiliated Hospital of Xinjiang Medical University from January 2008 to December 2016 provides compelling evidence that the incidence of mortality and cardiac death would be increased in patients with both hypoalbuminemia and high dNLR. To our knowledge, this is the first investigation of the association of ALB-dNLR with outcomes of patients who have undergone PCI.

The ALB-dNLR score measures both immune function and nutritional status. In this study, investigation of the possible association between the score and the clinical prognosis of post-PCI CAD patients demonstrated that (1) ALB-dNLR score was independently linked to adverse outcome in these patients, and (2) higher ALB-dNLR was significantly associated with an increased risk of all-cause death and cardiac death.

Serum albumin is reported to have both anti-inflammatory and immunomodulatory attributes (29), and reduced albumin synthesis caused by inflammation may have consequences for immune defense (30). Albumin levels are also influenced by nutritional status. Inflammation and malnutrition both reduce the albumin concentration by reducing its synthesis rate, while inflammation alone is associated with a higher catabolism rate, which expands nutrition by eliminating the defense mechanism that reduces the availability of protein and calories to protect the albumin pool. The adverse effects on body composition, and in extreme cases, the transfer of albumin from outside the vessel lumen, increases. What followed was a series of malignant events. Inflammation caused anorexia, reduced the effective use of protein and energy intake in the diet, and enhanced the catabolism of key albumin. The impact of inflammation on albumin levels was mainly related to low levels of albumin. Morbidity and mortality associated with albuminemia are related (31). The “TRIAGE” study found that increased inflammation indicators and increased malnutrition were independently associated with hypoalbuminemia. Low serum albumin levels, increased CRP values, and increased nutritional risk independently predicted 30-day mortality, and the area under the curve was 0.77, 0.70, and 0.75 (32). Inflammation and nutrition can promote the development of atherosclerosis (33). A meta-analysis found a 50% CAD risk with hypoalbuminemia (34). Low serum albumin levels also appear to be associated with increased CAD risk (35). A follow-up study of 8,750 AMI patients observed that low albumin levels on admission were associated with increased ACM (36).

Serum albumin was a significant parameter affecting GFR in addition to serum creatinine (37). GFR is not only a marker of the presence of conventional cardiovascular risk factors but may also have an important role in the pathogenesis of CVD, particularly CAD. GFR has numerous effects on the cardiovascular system, including inhibition of erythropoiesis and platelet function (38) and induction of volume overload (39), dyslipidemia, hypertension (40) and vascular calcification (41). In our study, we found that there were significant differences in eGFR among the three groups in baseline data (*P* = 0.038). However, the multivariable Cox regression model did not show that eGFR is an independent predictor of ACM, CM, MACCEs, and MACEs in post-PCI CAD patients. The dNLR is defined as the neutrophil count divided by the neutrophil count subtracted from the leukocyte count (18). The denominator includes a broad mix of monocytes and lymphocytes, which have different physiological roles. The dNLR is regarded as an indicator of inflammation and is used in a wide range of applications, including early cancer prediction, chemotherapy drug sensitivity, risk stratification of patients undergoing vascular surgery, distinction between non-alcoholic patients with severe fatty liver, and Alzheimer's disease. Earlier reports even suggested that it can be used as a diagnostic marker for appendicitis (42–45).

Inflammation is closely associated with atherosclerosis and cardiovascular disease (46, 47). Previous studies have shown that WBC counts are associated with CHD and an increased risk of ischemic disease (48). Leukocytes are key players in the vascular injury process, and total WBC counts are an indicator of the strength of this process. The Benjamin study (14) showed that total WBC counts proved to be an independent predictor of CAD, but neutrophil (N) or low lymphocyte (L) counts provided higher predictive value, with the best risk prediction given by the N/L ratio. Correlative studies have also shown (49, 50) that a higher NLR was associated with an increased risk of all-cause or cardiac death. In our study, the AUC of the ALB-dNLR+classic risk model was 0.794 (95% CI: 0.771–0.817), and the AUC of the dNLR+classic risk model was 0.720 (95% CI: 0.690–0.750). Therefore, we believe that the predictive performance of ALB-dNLR is stronger than that of dNLR alone. Compared to general WBC counts, ALB-dNLR has higher predictive power. The neutrophil to lymphocyte ratio represents the number of neutrophils in the body as a result of inflammation and is proposed as a new biomarker of systemic inflammation. The relative value of the number of lymphocytes produced by the “protection mechanism” was determined. Therefore, higher neutrophil and lymphocyte ratios represent inflammation, and inflammation can stimulate the production of neutrophils and accelerate the apoptosis of lymphocytes. Specifically, subacute inflammation in the body is important in cardiovascular disease and atherosclerosis (51).

In experimental atherosclerosis, the number of neutrophils in the blood increases and accumulates at an early stage of the injury. It helps special monocytes adhere or transport by releasing alarm proteins and other preformed granular proteins (52). Neutrophils also contain a large amount of myeloperoxidase, nicotinamide adenine dinucleotide phosphate (NADPH) oxidase and lipoxygenase, which contribute to oxidative stress, endothelial cell dysfunction, and disease growth and are the main determinants of instability.

Liu et al. (53) found that NLR is an independent risk factor for hospital mortality in COVID-19 patients (especially males). For every additional unit of NLR, the OR of male mortality after full adjustment was 1.10 (OR = 1.10; 95% CI: 1.02–1.19; *P* = 0.016). The study of NLR in predicting the long-term mortality of patients with non-ST-segment elevation myocardial infarction (NSTEMI) showed that after controlling for Global Registry of Acute Coronary Events risk profile scores, the average NLR level remained a significant predictor of inpatient and 4-year mortality. The hazard ratios per unit increase in the average NLR (log) increased by 1.06 (*p* = 0.0133) and 1.09 (*p* = 0.0006), respectively (54). Butt et al. (55) showed that a high NLR is an independent predictor of contrast-induced acute nephropathy in patients with acute myocardial infarction (AMI) (OR 2.03, 95% CI: 1.403–3.176, *P* <0.001).

These findings are consistent with our results. Our study found that ALB-dNLR was associated with the long-term mortality of CAD patients. Elevated ALB-dNLR may be an independent risk factor for long-term death in CAD patients who have undergone PCI. Compared with normal patients, patients with hypoalbuminemia and high dNLR have higher ACM and CM. However, the ALB-dNLR score does not seem to affect the incidence of MACCEs or MACEs. The ALB-dNLR reflects albumin and dNLR. Previous research has shown that low serum albumin predicted MACE only in patients with a low cardiovascular risk profile and was not associated with cardiac outcome in patients with 3 or more traditional cardiovascular risk factors (15). Our research included traditional cardiovascular risks such as smoking, hypertension, hyperlipidemia, and diabetes. Therefore, it can be explained that ALB-dNLR is associated with mortality but not MACE. In our study, we found that an ALB-dNLR score of 2 was significantly correlated for male but not female patients. Previous studies (56) have shown that premenopausal women are less likely to develop coronary heart disease than men, but post-menopausal women are more likely to develop coronary heart disease than men. The increased risk in women is largely attributable to the loss of sex steroids, especially estrogen. Estrogen can help regulate the metabolism of lipids in the body, maintain the function of endothelial cells and, to a certain extent, inhibit the proliferation of smooth muscle cells, help dilate blood vessels, and help prevent or delay the formation of atherosclerosis in the later stage. Our study also found that the results of this study have some clinical significance and advantages. The dNLR can be quickly calculated based on routine blood examination at the time of admission and the serum albumin obtained in the biochemical examination. Coronary angiography during hospitalization and medical history can be used to evaluate whether it is a chronic occlusive disease. Based on the results of angiography and ALB-NLR, the patient's health can be evaluated. The long-term prognosis and the need for readmission to the hospital later.

Study limitations include (1) due to the retrospective design of the study, the identified association should be considered hypothesis-generating; (2) mechanistic studies and prospective clinical studies supporting the prognostic value of ALB-dNLR score are lacking; and (3) the lack of hsCRP data makes it impossible to analyze the correlation between ALB-dNLR and hsCPR.

## Conclusions

ALB-dNLR is an independent risk factor of predict adverse outcomes in post-PCI CAD patients. Assessment of ALB-dNLR may help identify high risk individuals with CAD.

## What Is Known About This Topic?

- Both albumin (ALB) and derived neutrophil to lymphocyte ratio (dNLR) have been shown to be involved in the pathogenesis of coronary artery disease (CAD), chronic inflammatory disease and tumor.
- It has been reported that the ALB-dNLR score is an independent predictor for adverse outcomes of tumor.

## What Does This Paper Add?

- The present study indicates that ALB-dNLR score is an independent and novel predictor of adverse long-term outcomes in CAD patients who underwent PCI.

## Data Availability Statement

The original contributions presented in the study are included in the article/supplementary material, further inquiries can be directed to the corresponding author/s.

## Ethics Statement

The studies involving human participants were reviewed and approved by Ethics Committee of First Affiliated Hospital of Xinjiang Medical University. Written informed consent for participation was not required for this study in accordance with the national legislation and the institutional requirements.

## Author Contributions

W-JX, H-TY, and Y-YZ conceptualized the current study objectives, analyzed the data, and wrote the manuscript draft. T-TW, X-GH, and YY collected and organized data. XX and Y-TM had responsibility of the final content. All authors read and approved the final manuscript and were involved in the conception of the research plan.

## Funding

This research was funded by the National Natural Science Foundation of China (82170345).

## Conflict of Interest

The authors declare that the research was conducted in the absence of any commercial or financial relationships that could be construed as a potential conflict of interest.

## Publisher's Note

All claims expressed in this article are solely those of the authors and do not necessarily represent those of their affiliated organizations, or those of the publisher, the editors and the reviewers. Any product that may be evaluated in this article, or claim that may be made by its manufacturer, is not guaranteed or endorsed by the publisher.
